# Somatic CAG repeat instability in intermediate alleles of the *HTT* gene and its potential association with a clinical phenotype

**DOI:** 10.1038/s41431-024-01546-6

**Published:** 2024-03-04

**Authors:** Ainara Ruiz de Sabando, Marc Ciosi, Arkaitz Galbete, Sarah A. Cumming, Spanish HD Collaborative Group, Victoria Álvarez, Asunción Martinez-Descals, Montserrat Mila, Maria José Trujillo-Tiebas, Jose Luis López-Sendón, María Fenollar-Cortés, Inés Legarda, Sara Bernal Noguera, Jose M. Millán, Camen Durán-Herrera, Javier Ruiz-Martínez, Rebeca Ruiz Onandi, Darren G. Monckton, Maria A. Ramos-Arroyo

**Affiliations:** 1 Department of Medical Genetics, Hospital Universitario de Navarra, IdiSNA, 31008 Pamplona, Spain; 2 Department of Health Sciences, Universidad Pública de Navarra, IdiSNA, 31008 Pamplona, Spain; 3 Fundación Miguel Servet-Navarrabiomed, IdiSNA, 31008 Pamplona, Spain; 4 School of Molecular Biosciences, College of Medical, Veterinary and Life Sciences, University of Glasgow, Glasgow, G12 8QQ UK; 5 Department of Statistics, Informatics and Mathematics, Universidad Pública de Navarra, IdiSNA, 31006 Pamplona, Spain; 6 Department of Genetics, Hospital Universitario Central de Asturias, Oviedo, Spain; 7 Department of Neurology, Fundación Jimenez Diaz, Madrid, Spain; 8 Department of Biochemistry and Molecular Genetics, Hospital Clínic de Barcelona, Barcelona, Spain; 9 Department of Genetics and Genomics, Instituto de Investigación Sanitaria-Fundación Jiménez Díaz University Hospital, Universidad Autónoma de Madrid (IIS-FJD, UAM), Madrid, Spain; 10 Department of Neurology, Hospital Universitario Ramon y Cajal, Madrid, Spain; 11 Clinical Genetics Unit, Hospital Universitario Clínico San Carlos, Madrid, Spain; 12 Department of Neurology, Hospital Universitario Son Espases, Palma de Mallorca, Spain; 13 Biomedical Research Institute (IIB) Molecular Bases of Disease, Hospital de la Santa Creu i Sant Pau, Barcelona, Spain; 14 Laboratory of Molecular, Cellular and Genomic Biomedicine, Instituto de Investigación Sanitaria La Fe, Valencia, Spain; 15 Movement Disorders Unit, Hospital Universitario de Badajoz, Badajoz, Spain; 16 Department of Neurology, Hospital Universitario Donostia, San Sebastián, Spain; 17 Department of Pathology, Bioaraba Health Research Institute, Galdakao-Usansolo University Hospital, Galdakao-Usansolo, Spain

**Keywords:** Genetic predisposition to disease, Genetics research

## Abstract

Huntington disease (HD) is a neurodegenerative disorder caused by ≥36 CAGs in the *HTT* gene. Intermediate alleles (IAs) (27–35 CAGs) are not considered HD-causing, but their potential association with neurocognitive symptoms remains controversial. As *HTT* somatic CAG expansion influences HD onset, we hypothesised that IAs are somatically unstable, and that somatic CAG expansion may drive phenotypic presentation in some IA carriers. We quantified *HTT* somatic CAG expansions by MiSeq sequencing in the blood DNA of 164 HD subjects and 191 IA (symptomatic and control) carriers, and in the brain DNA of a symptomatic 33 CAG carrier. We also performed genotype-phenotype analysis. The phenotype of symptomatic IA carriers was characterised by motor (85%), cognitive (27%) and/or behavioural (29%) signs, with a late (58.7 ± 18.6 years), but not CAG-dependent, age at onset. IAs displayed somatic expansion that were CAG and age-dependent in blood DNA, with 0.4% and 0.01% of DNA molecules expanding by CAG and year, respectively. Somatic expansions of +1 and +2 CAGs were detected in the brain of the individual with 33 CAGs, with the highest expansion frequency in the putamen (10.3%) and the lowest in the cerebellum (4.8%). Somatic expansion in blood DNA was not different in symptomatic vs. control IA carriers. In conclusion, we show that *HTT* IAs are somatically unstable, but we found no association with HD-like phenotypes. It is plausible, however, that some IAs, close to the HD pathological threshold and with a predisposing genetic background, could manifest with neurocognitive symptoms.

## Introduction

Huntington disease (HD) (OMIM 143100) is a neurodegenerative disorder caused by the pathogenic expansion of the CAG trinucleotide in exon one of the huntingtin (*HTT*) gene. Alleles with ≥40 CAGs are fully penetrant, while alleles with 36–39 CAGs show reduced penetrance [1]. Alleles <36 CAGs are not considered HD-causing, although, those with 27–35 CAGs, known as intermediate alleles (IAs), can potentially expand into the disease range during germline transmission [2]. IAs are relatively common worldwide, with variable frequencies (from 0.45% in Thailand to 8.7% in Brazil) depending on the population [3]. IA carriers are highly unlikely to be formally diagnosed with HD; however, their potential association with an HD-compatible phenotype, including movement disorders, cognitive deterioration and/or behavioural changes [4–9], or their potential influence in other neurocognitive disorders [10–12], has been an area of great research and debate in the last decade.

The hallmark of an HD clinical diagnosis is the presentation of the classic motor symptoms. The age at motor onset is inversely correlated with the number of inherited CAG repeats [13], which accounts for ∼56% of its variability [14]. Some of the variability not explained by inherited CAG length has mainly been attributed to DNA repair gene variants [15], some of which have also been associated with the somatic instability of the *HTT* repeat in blood DNA [16]. Altogether, the latest data suggest that the timing of HD onset is determined by somatic expansion of the CAG repeat beyond its inherited length [15, 17, 18] in a disease model that involves two subsequent events: first, the inherited *HTT* CAG repeat length somatically expands until a harmful threshold is reached, to then trigger a process of cellular degeneration.

Somatic mosaicism of the *HTT* CAG repeat is the consequence of postzygotic variants that lead to differences in the number of CAGs in cells and tissues of the same individual. In HD patients, higher levels of somatic expansion of the *HTT* CAG repeat in blood DNA are associated with earlier disease onset and more severe HD pathogenesis [16, 19]. Although this has not yet been reported, it is probable that intermediate *HTT* alleles, like HD-causing alleles, are somatically unstable. If that is indeed the case, cells more susceptible to expansion and critical for HD pathology, i.e., striatal cells, might see their *HTT* CAG repeat cross the pathological HD threshold [20]. If present, these larger expansions might be responsible for the neurocognitive symptoms observed in some IA carriers [7–9].

We hypothesised that *HTT* IAs are somatically unstable, and that somatic expansions may drive phenotypic presentation in some IA carriers. To test these hypotheses, we studied the degree of somatic CAG expansions in a large intermediate and expanded allele cohort and explored the CAG length and age effects in different allele categories. We further analysed the neurocognitive phenotypes of a large cohort of IA carriers, and studied the brain of a 33 CAG allele carrier that presented with neurocognitive signs.

## Materials and methods

### Study population

Study subjects were identified among individuals with HD-compatible symptoms, referred at 11 Spanish HD diagnostic laboratories to confirm or rule out this disease, and a cohort from the general population, as previously described [21]. For this study, we ascertained 490 HD cases and family members, from one HD reference centre, Hospital Universitario de Navarra (HUN), and 191 IA carriers, including those from the general population, identified by all 11 HD centres. Available clinical, familial and age at sampling data were also collected.

Sequencing studies were conducted in a subset of 355 blood DNA samples from ≥27 CAGs *HTT* carriers. Phenotype studies were performed in HD subjects and IA carriers with available clinical data (see Supplementary Fig. 1 for details). IA carriers with HD family history were further studied. Clinical and family data of four symptomatic IA carriers were thoroughly revised. Postmortem brain tissues of one of them were also analysed.

### *HTT* CAG repeat sizing by capillary electrophoresis

All DNA samples were re-analysed in one centre (HUN). *HTT* CAG length was determined as previously described [21].

### *HTT* exon one sequencing

The *HTT* exon one repeat region was amplified from 20 ng of peripheral blood DNA of 355 carriers of ≥27 CAG alleles, as estimated by capillary electrophoresis. We also analysed the DNA from frozen tissue of ten postmortem brain regions of a 33 CAG repeat symptomatic carrier.

PCR libraries were generated using MiSeq System-compatible primers and MiSeq sequencing was performed as previously described [22], using a 400 nt forward read and a 200 nt reverse read by Glasgow Polyomics (https://www.polyomics.gla.ac.uk). MiSeq reads were analysed with ScaleHD (v1.1 https://pypi.org/project/ScaleHD/). Alleles were genotyped for their inherited number of CAG repeats, as previously described [16].

For each allele, we quantified the number of variants up to 10 CAGs longer than the inherited allele using the measure $\frac{{{\sum }_{i=1}^{10}{n}}_{+i}}{n}$ (referred to as ‘ratio of CAG expansions’ in the rest of the manuscript), where $n$ is the number of MiSeq reads corresponding to the inherited allele, and ${n}_{+i}$ is the number of MiSeq reads corresponding to the sequenced variants with $i$ more CAG repeats than the inherited allele.

Within a DNA sample, when both the short and long alleles have the same (CAACAG)_(CCGCCA)_(CCG) allele structure, backward PCR slippage products from the longer allele have the potential to interfere with the accurate quantification of somatic expansions. To avoid this potential interference, alleles used for somatic expansion studies were selected according to the criteria detailed in Supplementary Methods.

For the brain DNA study, in addition to *HTT* exon one sequencing as described above, we determined the proportion of products larger than a 33 CAG inherited molecule generated by PCR, in the absence of somatic expansions. For that, MiSeq libraries were prepared with a lower quantity of input DNA, so a high percentage of reactions contained only a single 33 CAG input molecule. The 33 CAG cerebellum DNA sample (the brain tissue with the lowest frequency of expansions) was diluted to reach 5 pg input DNA for the MiSeq library preparation PCRs.

### Small-pool PCR

To identify the potential presence of large CAG somatic expansions, we performed small-pool PCR in blood and brain (hippocampus, cerebellum, medulla, pons, substantia nigra, frontal lobe, occipital lobe, temporal lobe, caudate nucleus and putamen) DNA samples, as previously described [23]. We amplified 300 pg template DNA using the locus-specific primers F 5’-ATGGCGACCCTGGAAAAGCTGATGAA-3’ and R 5’-GGCGGCTGAGGAAGCTGAGGA-3’. The amplified products were loaded on 1.5% agarose gel and Southern blot hybridised using a CAG•CTG repeat probe.

### Gene panel sequencing

Three HD family members, symptomatic carriers of an IA, were tested for other 51 HD-like genetic neurodegenerative diseases (Supplementary Table 1).

Complete exome libraries were generated with KAPA HyperCap (Roche) and sequencing was performed on NovaSeq 6000 (Illumina) with a read length of 150 nt. For variant identification, the genomic data were analysed using the DRAGEN Bio-IT Platform (Illumina), with the DRAGEN Germline pipeline. The repeat lengths were determined based on the DRAGEN ExpansionHunter (Illumina). The reference genome was GRCh37d5. Variants were selected and filtered with Variant Interpreter (Illumina).

### Postmortem neuropathologic brain studies

Morphologic brain studies were performed on a 33 CAG repeat carrier that presented HD-like signs, who died at the age of 69 of colon cancer. Selected areas underwent neuropathological examination by studying dewaxed sections stained with haematoxylin-eosin, or different antibodies for immunohistochemistry: β-amyloid (clone 12F4 monoclonal antibody), phosphorylated tau (P-tau) (AT8 MAb, sp409/410 monoclonal antibody), α-synuclein (amino acids 115–121, clone LB509 monoclonal antibody), TDP-43 (sp409/410 monoclonal antibody) and p62 (Monoclonal purified mouse Anti-p62 LCK ligand clone 3/P62). In addition, polyQ, P62 and ubiquitin analysis were performed in sections from frontal lobe, caudate nucleus, anterior thalamus and posterior hippocampus, searching for intranuclear inclusions. These techniques were performed in two different centres (Hospital Universitario de Alava and Hospital Clinic Barcelona) and analysed by two independent trained pathologists.

### Statistics

Statistical analysis was performed using IBM SPSS Statistics (Windows, v20.0). To study the association of CAG length and age with the ratio of CAG expansions, linear regression analysis of 10 to 50 CAG alleles, grouped into normal, intermediate, reduced penetrance and full penetrance categories, were conducted, assuming linearity in each group. Results on age at onset and type of symptoms were summarised using descriptive statistics. The differences in mean age at onset for each CAG length group were determined using Student’s *t* test. All tests were two-sided and a *p* value less than 0.05 was considered statistically significant.

## Results

### Somatic expansions are CAG- and age-dependent in IAs in peripheral blood

**Fig. 1:**
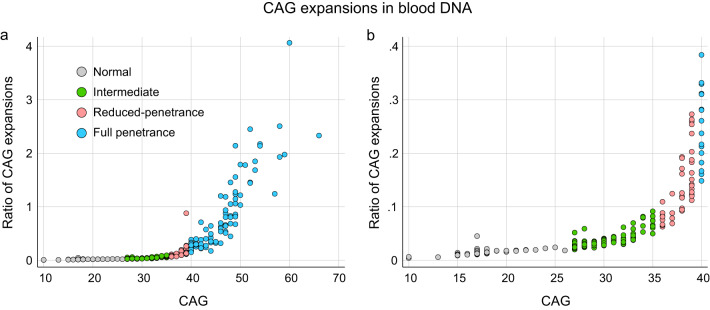
Somatic expansions in *HTT* alleles by CAG length. The ratio of CAG expansions (number of somatic expansion sequenced reads/number of inherited allele sequenced reads) relative to the number of CAGs from the inherited allele in peripheral blood is depicted for (**a**) allele sizes ranging from 10 to 66 CAGs (*n* = 337), and (**b**) a closer view for allele sizes ranging from 10 to 40 CAGs.

**Fig. 2:**
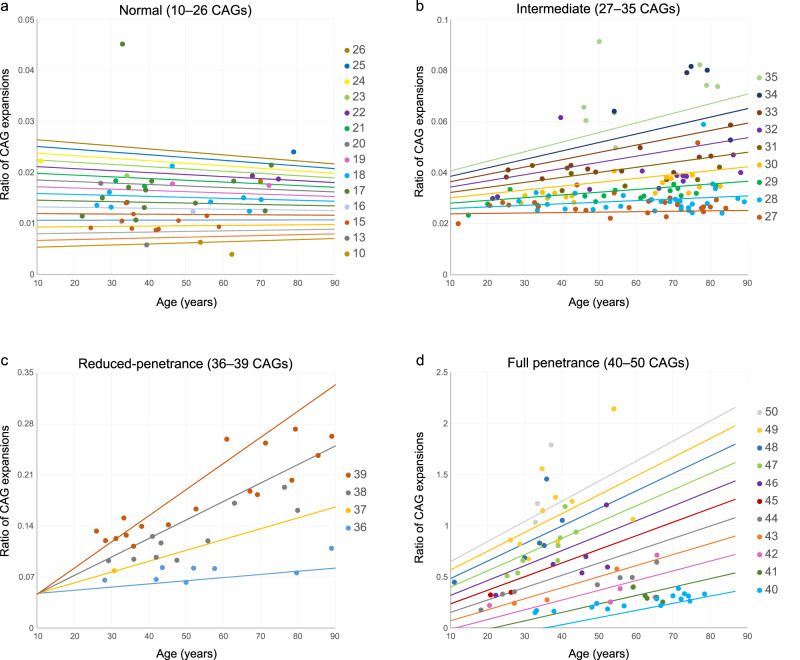
The effect of CAG length and age in the somatic expansions of *HTT* alleles. The graphs show the ratio of somatic expansions (number of somatic expansion sequenced reads/number of inherited allele sequenced reads) in peripheral blood plotted against the age of the carriers of (**a**) 47 normal, (**b**) 135 intermediate, (**c**) 37 reduced-penetrance and (**d**) 74 full penetrance alleles. Each data point is coloured with respect to the CAG length of the inherited allele. The scatterplots also present the linear regression lines for each CAG repeat length, which include the interaction between age at sampling and CAG repeat length.

To determine if IAs are somatically unstable, we used high-throughput ultra-deep MiSeq sequencing to analyse the *HTT* repeat in a large cohort of 355 individuals, including 164 and 191, disease-associated alleles and IA carriers, respectively. The ratio of CAG expansions could be investigated in 337 chromosomes with inherited alleles ranging from 10 to 66 CAG (Fig. 1). We observed that the proportion of CAG expansions are CAG-length dependent for all CAG ranges, most notably for larger alleles, and age-dependent in IAs and expanded alleles (Fig. 2 and Table 1). More specifically, in our cohort, the age effect reaches statistical significance in alleles of CAG ≥ 29 (Supplementary Table 2). This age-dependence of the measured proportion of CAG expansions demonstrates that these observed CAG expansions are in fact somatic expansions. For all IAs (*n* = 135), the ratio of CAG expansions increased by 0.004 per CAG (0.4% expanded DNA molecules) and by 0.0001 per year (0.01% expanded DNA molecules) (Table 1). Additionally, they showed a significant positive interaction between CAG and age effect, i.e., the higher the age, the higher effect of CAG on the proportion of CAG expansions (*p* = 0.01). Alleles of >35 CAGs showed greater increases in CAG expansions per extra CAG unit and year, with 0.047 and 0.003 for reduced-penetrance alleles (*n* = 37), respectively, and 0.133 and 0.011 for full penetrance alleles (*n* = 88), respectively.

**Table 1:** Linear regression results showing the main effects of the number of CAGs in the inherited allele and age at sampling on the ratio of CAG expansions for alleles ranging from 10 to 50 CAGs, categorised into normal, intermediate, reduced-penetrance and full-penetrance alleles.

Allele type (CAG range)	*n*	Min	Max	Ratio of CAG expansions^a^							
				Main effects							Interaction effects
				CAG			Age			*R*^2^	
				*β*-coefficient	CI (95%)	*p* value	*β*-coefficient	CI (95%)	*p* value		*p* value
Normal (10-27)	47	0.004	0.024	0.001	0.001, 0.002	<0.001	−0.00002	−0.0001, 0.0001	0.719	0.324	0.736
Intermediate (27-35)	135	0.020	0.091	0.004	0.004, 0.005	<0.001	0.0001	0.0001, 0.0002	0.001	0.646	0.010
Reduced-penetrance (36-39)	37	0.063	0.878	0.047	0.014, 0.079	0.006	0.003	0.001, 0.005	0.009	0.332	0.200
Full penetrance (40-50)	74	0.148	1787	0.125	0.107, 0.143	<0.001	0.011	0.007, 0.015	<0.001	0.742	0.031

^a^The proportion of reads with more CAGs than the inherited CAG length.

### Symptomatic IA carriers do not show increased somatic CAG expansions or CAG-dependent onset of signs compared to population controls

IA carriers were categorised into population controls (*n* = 46), symptomatic (*n* = 78) and unknown phenotype (*n* = 9) (Supplementary Fig. 2). The ratio of CAG expansions adjusted by CAG length and age was not significantly different between population control and symptomatic IA carriers (0.038 vs. 0.035, *p* = 0.118).

**Fig. 3:**
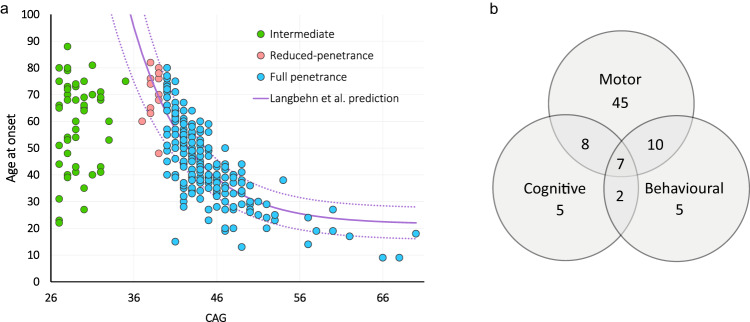
Neurocognitive phenotype of *HTT* intermediate alleles carriers. The graphs summarize the clinical characteristics of symptomatic cases represented as, (**a**) Onset of symptoms in intermediate (green, *n* = 49), reduced-penetrance (orange, *n* = 13) and full-penetrance (blue, *n* = 229) alleles carriers by the number of CAG repeats. The purple lines indicate the prediction of age at onset by Langbehn et al. [50], average (solid line) ± SD (dotted lines). (**b**) Venn diagram depicting the breakdown of neurocognitive signs into motor, cognitive and behavioural in 82 *HTT* intermediate allele carriers.

Available clinical data on the neurocognitive signs in IA carriers is presented in Supplementary Table 3. Symptomatic IA carriers presented with motor (85%), cognitive (27%) and/or behavioural (29%) signs (*n* = 82) (Fig. 3b). The average age at onset was 58.7 ± 18.6 years (*n* = 49), significantly lower than that observed for reduced penetrance alleles (68.3 ± 11.2 years, *n* = 13; *p* = 0.021) and significantly higher than full penetrance alleles (45 ± 14.1 years, *n* = 229; *p* < 0.001). Similar results were obtained for individuals with motor symptoms (58.8 ± 17.2 years, *n* = 38). When age at onset of IAs and HD cohorts were plotted according to their CAG length (Fig. 3a) we observed that clinical onset in IA carriers did not show a CAG-dependent correlation in contrast with reduced and full penetrance allele carriers. Interestingly, our cohort of symptomatic IAs follows a distribution similar to that of the tail of the distribution in the general population, with decreasing frequencies as the IA CAG number increases (i.e. 27 CAGs > 28 CAGs > 29 CAGs, etc.).

### Symptomatic IA carriers in HD families

Four clinically well-characterised male IA carriers, members of three HD families (Supplementary Fig. 3), presented a variety of HD-like neurological signs.

#### Family A

The proband, brother of a 43 CAG carrier, presented with progressive memory loss, lack of concentration, behavioural and motor alterations that initiated a few years after he suffered from a left occipitotemporal cerebral infarction at 54 years. Behavioural changes consisted of irritability, verbal aggressiveness and perseverative thinking. Neuropsychologic evaluation demonstrated severe difficulties to remember and learn new verbal material, and deterioration of executive functions. He presented with a slow and wide-based gait, in the absence of choreic movements. He carried a 33 CAG allele, sharing the familial extended *HTT* haplotype (A3a). He died at the age of 69 of colon cancer, 1 year after diagnosis. No family history of cancer was referred.

Postmortem brain studies demonstrated slight atrophy in the temporal, frontal and parietal lobes, and the head of the caudate nucleus showed a depression in its upper quarter. There was also a cavitated lesion in the lenticular nucleus along with atheromatous plaques in the circle of Willis and in temporal cerebral arteries, which partially occluded their lumen by up to 40–50%. Microscopically, the depression in the caudate nucleus corresponded to subacute ischaemia with abundant reactive astrocytes. Other microscopic findings included multiple infarcts, thalamic gliosis, small-vessel injury, and iron deposit in the lenticular and caudate nuclei. Similar vascular lesions were also present in the frontal lobe and the hippocampus.

Immunohistochemical techniques showed positive staining for AT8, in the form of threads, pretangles and tangles in the anterior hippocampus. Results were negative for amyloid and α-synuclein proteins, as well as for nuclear or intracytoplasmic HTT inclusions.

**Fig. 4:**
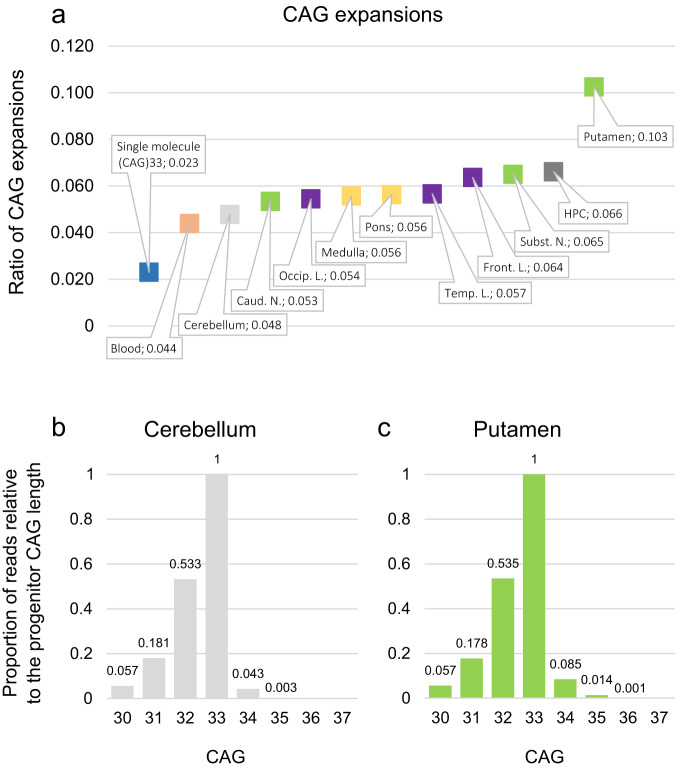
Somatic instability of the CAG repeats of a 33 CAG allele carrier. Somatic expansion ratio in single 33 CAG molecule data, showing the proportion of expansions generated by PCR (only +1 CAG) (blue), and in peripheral blood (orange) and brain tissues (**a**). The proportion of reads relative to the inherited CAG length in the cerebellum (**b**) and putamen (**c**). Caud N caudate nucleus, Occip L occipital lobe, Temp L temporal lobe, Front L frontal lobe, Subst N substantia nigra, HPC hippocampus.

MiSeq sequencing of brain DNA samples revealed somatic expansions of +1 and +2 CAGs in ten different brain tissues. The highest ratio was observed in the putamen, where up to 10.3% of the DNA molecules underwent expansion (Fig. 4a), and the lowest in cerebellum (4.8%), similar to peripheral blood (4.4%). Expansions of +1 and +2 CAGs were 2-fold and 5-fold higher, respectively, in putamen compared to cerebellum (Fig. 4b, c). In the single molecule experiment (i.e. in the absence of somatic expansions in the template DNA used for PCR), the only PCR products larger than the 33 CAG molecule contained 34 CAGs and their proportion relative to the 33 CAG products was 2.3%. Thus, in the absence of somatic expansions, our assay would yield a ratio of CAG expansion around 0.023 for 33 CAG alleles. The ratio of CAG expansion obtained for all the brain tissues was well above 0.023 (Fig. 4a), demonstrating the presence of genuine somatic expansions. Small-pool PCR analysis did not demonstrate CAG length variability different than that observed with sequencing (Supplementary Fig. 4).

#### Family B

The proband was a 73-year-old male, carrier of a 32 CAG allele (haplotype A2a) that expanded into the pathological range in his son. For the last 5 years, he presented memory problems and apathy, followed by executive dysfunction, temporal disorientation and generalised weakness associated with lower motor neuron lesion, based on electromyographic studies. No behavioural or psychiatric signs were observed. He referred a paternal positive family history of dementia of late-onset.

#### Family C

The proband, carrier of a 32 CAG allele, presented bilateral dystonic movements of the upper extremities, predominantly of the right side, that initiated at an early age. He was last evaluated at the age of 55, showing dystonia with myoclonic component, dysmetria and blepharospasm. Dystonia also affected the neck and larynx, sparing the lower extremities. He had no difficulties with walking, cognition or mood. The father was evaluated at the age of 73 years, showing mild walking difficulties in the absence of involuntary movements or definitive behavioural or cognitive signs. He carried a 33 CAG allele (haplotype C1) that expanded to 42 CAGs in another descendent. Notably, this allele was atypical in structure lacking the entire CAACAGCCGCCA intervening sequence between the pure CAG repeat and the CCG tract (Human Genome Variation Society [HGVS] nomenclature LRG_763t1:c.[118A > G;127A > G]).

Analysis of the selected gene panel sequencing did not identify pathogenic/likely pathogenic variants or expansions in the probands of these three families.

## Discussion

HD is one of about 50 disorders caused by the expansion of simple sequence repeats. In triplet repeat disorders, expanded disease-associated alleles are, in contrast with normal-length alleles, genetically highly unstable [16, 24–26]. However, some larger normal alleles have also been shown to be genetically unstable and at risk of expanding into the disease range during germline transmission. Different terminology has been applied to such alleles, being described as ‘premutation’ alleles at the *FMR1* (55–199 repeats) and *DMPK* loci (~40–50 repeats), or ‘intermediate’ alleles at the *HTT* locus (27–35 repeats). In addition, some alleles just below the disease threshold or the premutation range have been associated with milder and/or differential phenotypes in several loci (*FMR1*, *ATXN1*, *ATXN2*, *C9orf72*) [27–31], and this range is referred to as the ‘IA‘, ‘uncertain significance’ or ‘grey zone’.

*HTT* IAs have been defined due to their capacity to expand into the HD range in the germline [2], and, by definition, are not considered HD-causing. However, multiple reports have described associated neurocognitive symptoms in some IA carriers [4–12], opening this matter to further research. Symptomatic IA carriers represent a small fraction of the IA carriers in the general population (5–6% in Caucasian populations) [21, 32], a circumstance that greatly hinders their investigation. Our study, which includes the largest published cohort of symptomatic *HTT* IA carriers systematically ascertained, aims to analyse their clinical characteristics and to investigate the role of somatic expansions of *HTT* IAs as a modifying factor of the phenotype.

We first evaluated the possible genotype-phenotype correlation, under the hypothesis that IA carriers might represent a milder end of the phenotypic spectrum of *HTT* expansions. Most cases of the study showed motor signs (85%) and/or cognitive and/or behavioural difficulties (45%), and a late onset of symptoms. However, the mean age at onset was lower in IAs (58.7 ± 18.6 years) than in reduced-penetrance carriers (68.3 ± 11.2 years). Over 40% of cases manifested before the age of 60 and the age at onset did not correlate with the number of CAGs, neither in our data nor in IA cases of previous literature reports [4, 7, 33–40] (Supplementary Fig. 5). Therefore, it seems unlikely that the potential *HTT-*associated phenotype of IA carriers, if any, represents the mildest end of an HD continuum. It is worth noting, however, that HD family C was segregating an atypical 33 CAG IA/42 CAG allele in which the CAA repeat interruption was lost, a structure associated with an earlier onset and/or more severe disease course [15, 16, 41]. A previous analysis of variants and frequencies of this intervening sequence showed that only 1% of IAs in our cohort carry the CAA deletion LRG_763t1:c.118A > G [21]. It is thus possible that, in some IA carriers, a specific combination of predisposing environmental and genetic risk factors, other than CAG length, have resulted in an extremely early onset or a reduction of the penetrance threshold for an HD-like phenotype.

Much evidence has now accumulated that somatic expansion is a key driver of disease pathology. In HD, full penetrance HD alleles are somatically unstable in blood DNA, in a process that is highly expansion-biased and age-dependent, and associated with variation in age at onset, disease progression and pathology [16, 18, 42]. In the plausible scenario that extreme somatic expansion might cause clinical signs in IA carriers, we analysed somatic CAG expansion ratios in alleles ranging from 10 to 50 repeats. We demonstrated that instability in blood DNA is not only present in full penetrance alleles, but it also extends to reduced-penetrance and intermediate alleles, with somatic gains of +1 to +3, and +1 to +2 CAG repeats, respectively, compared to the number of repeats of the inherited allele. As observed in Fig. 1, our results emphasise the concept of the continuum of somatic expansion observed at the *HTT* locus, rather than the somewhat arbitrary, but convenient, clinically defined categories (normal, intermediate, reduced-penetrance, full-penetrance). Nevertheless, we did not reveal an increase in the level of somatic expansion in blood DNA of symptomatic IA carriers versus IA population controls, although we cannot rule out the presence of more somatic expansions in the brains of some symptomatic IA carriers.

Tissue-specificity of somatic expansions has been reported in HD, as well as in other triplet repeat diseases [26, 43]. Brain cells of HD individuals show a much higher degree of CAG instability compared to peripheral tissues, being especially striking in the striatal medium spiny neurons [25, 44]. We studied the brain of a 33 CAG carrier, with an HD family history, presenting cognitive difficulties, irritability and mild motor alterations, and analysed the presence of somatic expansions in several brain regions that might contribute to this phenotype. As previously observed for expanded alleles, we here demonstrate the tissue-specificity of somatic expansions in IA carriers, with relatively lower somatic expansion rates in blood compared to other brain tissues. Our patient showed the highest ratio in the putamen (10.3%), and the lowest in the cerebellum (4.8%), a somatic expansion pattern similar to that observed in the early-stage brain of HD [25, 45]. Nevertheless, these expansions were only in the order of +1 to +2 CAG repeats, and small pool-PCR analysis did not reveal the presence of rarer large-length change events. It seems, therefore, unlikely, that this mechanism was sufficient to reach the established pathological repeat threshold for inherited alleles, let alone the hypothesised threshold for cellular pathology [17, 24], that might explain the phenotype in this case.

Anatomopathological brain studies in our patient identified iron deposits, as well as atrophy and several lesions, affecting the frontal lobe, the caudate nucleus and the hippocampus; however, these findings seemed largely attributable to the vascular injury, a circumstance that could mask the potential effect, if any, of other associated pathogenic mechanisms. We did not identify intranuclear inclusions or protein deposits in the examined sections, except for the presence of abnormally phosphorylated tau, which, also, may well be secondary to chronic vascular damage. We are aware of seven additional postmortem brain studies of symptomatic patients with *HTT* IAs. Two patients with 28 and 29 CAGs, clinically diagnosed with HD, showed caudate and putamen atrophy and one of them had minimal inclusions [4, 8]. In a recent study [12], three of five *HTT* IA carriers, with clinical and neuropathological diagnoses of multiple system atrophy parkinsonian type, presented striatonigral degeneration, and two of them had polyglutamine staining in isolated neurons in pons and basal nuclei. These clinico-anatomopathologic studies, although important to, eventually, unravel the complex etiopathologic mechanism of HD, do not allow us to draw definite conclusions on the genotype-phenotype correlation of *HTT* IAs.

Interestingly, however, there seems to be increasing evidence of the contribution of intermediate and pathogenic repeat alleles to other neurological diseases, not associated to the classic phenotype of the gene expansion. IAs in the *ATXN2* and *ATXN1* genes may cause amyotrophic lateral sclerosis [28, 29], and grey zone alleles of the *FMR1* have been associated with parkinsonism [27]. With respect to HD, an apparently significantly higher frequency of expanded and intermediate CAG repeats has been described among patients with Alzheimer’s disease, frontotemporal dementia (FTD) and multiple system atrophy [10–12]. Whole genome sequencing analysis of a large sample of subjects with FTD and amyotrophic lateral sclerosis (ALS) also identified patients with pathogenic *HTT* expansions and postmortem neuropathologic features of ALS, suggesting a contribution to the phenotype [46]. Although differential diagnosis of HD versus FTD or other neurodegenerative disorders can sometimes be very challenging [47], it is also possible that different repeat expansion disorders share common underlying mechanisms that could result in unique or overlapping neurological symptoms, which might also depend on the length of the repeats.

Unfortunately, clinical information on most symptomatic IA carriers in our study is not sufficiently specific and robust to delineate a specific associated neurological disorder. Neither could we demonstrate a significantly higher frequency of IAs among symptomatic patients with respect to population controls (3.7% vs. 2.5%, respectively, *p* = 0.06; data not shown). Given the relatively high proportion of IA carriers in the population, it seems possible that, symptomatic IA carriers may suffer, by chance, from other neurological diseases. It is worth noticing, however, that nearly 50% of IA carriers presented neurocognitive and psychiatric signs; two of the three members of HD families more thoroughly investigated showed signs of dementia, mood disorders and/or parkinsonism, and pathological variants, including repeat length diseases, in another 51 HD-like associated genes were ruled out. Similarly, previous studies have reported a high proportion of HD-negative family members showing neuropsychiatric signs [48, 49]. Whether it actually reflects susceptibility to neurological diseases and/or disease burden, is not yet known. More recently, prospective longitudinal HD studies with large cohorts found a link between IA carriers and behavioural abnormalities [5], or a greater cognitive decline compared to controls [6]. It is therefore plausible that high CAG repeats in the IA range of the *HTT* gene might confer susceptibility to the development of different neurodegenerative disorders, perhaps secondary to the common etiopathological pathways. In order to confirm this hypothesis further large longitudinal studies and additional clinical-anatomopathological analysis on *HTT* IA carriers will be needed.

In conclusion, we cannot demonstrate that *HTT* IAs can trigger a mild HD phenotype, following a CAG-dependent symptomatic continuum. In contrast, we do confirm that the somatic expansion ratio follows a gradual CAG correlation from expanded to reduced-penetrance and intermediate alleles, which is also age-dependent and tissue-specific. The low level of repeat instability observed in blood DNA in IAs cannot be considered a major modifying factor of the phenotype in these patients. However, it may be possible that some *HTT* IAs, especially those closer to the HD pathological threshold and with a predisposing genetic background, manifest with HD-compatible symptoms. Additionally, it is also likely that IAs might act as one of multiple susceptibility factors to develop other neurocognitive diseases, through a common pathogenic mechanism shared with other repeat expansion disorders.

### Supplementary information

Supplementary material

## Data Availability

Study protocol and deidentified raw data sets generated and/or analysed during this study will be available upon request. Any individual personal/clinical data will not be available.
