# Supplementary material for: Somatic CAG repeat instability in intermediate alleles of the HTT gene and its potential association with a clinical phenotype
Source: Eur J Hum Genet. 2024 Mar 4;32(7):770–8. doi: 10.1038/s41431-024-01546-6 (PMC11220145; doi:10.1038/s41431-024-01546-6)
Supplement: Supplementary file 1 — Supplementary material [file 41431_2024_1546_MOESM1_ESM.pdf]

SUPPLEMENTARY MATERIAL

"Somatic CAG repeat instability in intermediate alleles of the HTT gene and its potential association with a clinical phenotype"

1. Supplementary Methods

1.1. *HTT* exon one sequencing. Allele selection criteria.

Within a DNA sample, when both the short and long alleles have the same (CAACAG)_(CCGCCA)_(CCG) allele structure, backward PCR slippage products from the longer allele have the potential to interfere with the accurate quantification of somatic expansions. To avoid this potential interference, we have only quantified the ratio of somatic expansions on the long allele when both alleles had the same (CAACAG)_(CCGCCA)_(CCG) allele structure and the difference between both alleles was >5 CAGs. When the two alleles had different (CAACAG)_(CCGCCA)_(CCG) allele structures, the ratio of somatic expansions was quantified on both alleles if the difference in the number of CCG repeats was >1 CCG between the short and long *HTT* allele or if the two alleles had different (CAACAG)_(CCGCCA) sequences. The total number of alleles for which the ratio of CAG expansions was quantified was 337 (*i.e.* the long allele in 257 samples and both alleles in 40 samples). This ratio was calculated with the following equation: $\frac{number\ of\ somatic\ expansion\ reads}{number\ of\ inherited\ allele\ reads}$.

2. Supplementary Tables

2.1. **Supplementary Table 1** List of genes with the 51 HD-like neurodegenerative diseases that were analysed for three symptomatic carriers of IAs, based on their individual phenotypes.

Variant type	List of genes
Pathogenic Single Nucleotide Variants, Muti-Nucleotyde Variants, insertions, deletions, and indels.	*APP*, *ATP13A2*, *ATP7B*, *C19orf12*, *CHCHD10*, *CHMP2B*, *CP*, *DCAF17*, *DCTN1*, *FA2H*, *FTL*, *FUS*, *GNAL*, *KMT2B*, *HPRT1*, *KCTD17*, *MATR3*, *NKX2-1*, *NOTCH3*, *NUP62*, *OPTN*, *PANK2*, *PDE10A*, *PDE8B*, *PDGFRB*, *PLA2G6*, *PRNP*, *PSEN1*, *PSEN2*, *REPS1*, *RNF216*, *SGCE*, *SLC20A2*, *SQSTM1*, *TARDBP*, *TBK1*, *TBP*, *TOR1A*, *UBQLN2*, *VAC14*, *VCP*, *VPS13A*, *WDR45*, *XK*
CAG repeat expansions	*ATN1*, *ATXN1*, *ATXN2*, *ATXN3*, *ATXN7*, and *JPH3*
GGGGCC repeat expansions	*C9orf72*

2.2. Supplementary Table 2 The effect of age in the somatic instability of *HTT* intermediate alleles. The linear regression analysis for each individual CAG length in the intermediate range, which include CAG length and age main effects, show that somatic expansions in alleles ≥29 CAGs are age-dependent. It is worth considering that the sample size is a limitation for some CAG length groups that do not reach statistical significance in our cohort, but the general trend, when plotted all together (see **Fig. 2**) confirms the change at the 29 CAGs.

**CAG**	**n**	**p-value (age)**	**R^2**
27	25	0.328	0.042
28	31	0.467	0.018
29	17	0.002	0.476
30	18	0.003	0.440
31	8	0.896	0.003
32	12	0.507	0.045
33	12	0.009	0.509
34	4	0.109	0.793
35	8	0.389	0.126

2.3. Supplementary Table 3 Clinical data collected retrospectively from 89 intermediate allele carriers from 11 different centres in Spain. We used the clinical and family data described by the referring neurologist at genetic testing, and searched for further information by reviewing the clinical charts or additional medical reports, when available.

N°	Allele 1	Allele 2	Sex	Age at onset	Age at clinical diagnosis	Neurocognitive phenotype		
						**Motor signs**	**Cognitive signs**	**Behavioural signs**
**1**	27	16	F	23	24	Generalised choreic movements	No	No
**2**	27	16	U		58	Choreic movements	Cognitive decline	No
**3**	27	16	F	80	81	Choreic movements	No	No
**4**	27	17	F	66	70	Choreic movements	No	No
**5**	27	17	F	44	46	*not available*		
**6**	27	17	M		78	Choreic movements	No	No
**7**	27	17	F		78	Choreic movements	Cognitive decline	No
**8**	27	17	U		84	Choreic movements	Dementia	No
**9**	27	17	M		53	No	No	Psychiatric disease, anxiety
**10**	27	17	F	75	80	Unspecified motor symptoms	No	Depression
**11**	27	18	U		78	Choreic movements	No	No
**12**	27	18	U		63	Choreic movements	No	No
**13**	27	19	F	65	67	Choreic movements	No	Psychiatric disease
**14**	27	20	F			No	Memory loss, speech and comunication difficulties	Behavioural disorder, apathy
**15**	27	22	F		68	No	Dementia	No
**16**	27	23	U		77	Choreic movements	No	No
**17**	27	24	M	31	40	Choreic movements	No	No
**18**	27	25	F	22		Hyperkinesia	Unspecified cognitive signs	Compulsive behaviour, boderline personality disorder
**19**	27	25	M	51	54	No	Cognitive decline	Anxiety, depression
**20**	28	10	U		36	Involuntary movements	No	No
**21**	28	14	U		72	Hemichorea	No	No
**22**	28	15	M	73	74	Choreic movements	No	Psychiatric disease, hallucinations
**23**	28	15	U		74	Generalised choreic movements	No	No
**24**	28	16	F	70	72	*not available*		
**25**	28	17	F	53	57	Dystonia	No	No
**26**	28	17	F	80	87	No	Dementia	Depression
**27**	28	17	M		81	Involuntary movements	No	No
**28**	28	17	M	48	55	Ataxia	No	No
**29**	28	17	M		66	Oro-bucal dyskinesia	Cognitive decline	Anxiety, depression
**30**	28	17	U		74	Choreic movements	No	No
**31**	28	17	U		83	Choreic movements	Cognitive decline	No
**32**	28	17	U			Choreic movements	No	No

**33**	28	18	U		64	Choreic movements	No	No
**34**	28	18	F	88	89	*not available*		
**35**	28	19	M	68	73	Choreic movements	No	No
**36**	28	19	F	39	72	Oro-bucal diskinesia	No	No
**37**	28	20	F	79		Involuntary movements, parkinsonism	No	No
**38**	28	20	F		64	Oro-bucal diskinesia	No	No
**39**	28	20	F	54	79	Choreic movements	No	Depression
**40**	28	20	F		71	Choreic movements (head)	No	Psychiatric disease
**41**	28	21	M	40	43	Involuntary movements	No	no
**42**	28	22	M	40	40	*not available*		
**43**	28	23	U		72	Choreic movements, oro-bucal dyskinesia	No	No
**44**	28	23	M	72	73	Choreic movements	No	No
**45**	29	15	M	54	64	Involuntary movmentes, parkinsonism	No	No
**46**	29	17	F	74	76	Facial chorea	No	No
**47**	29	17	F	68	71	Choreic movements, ataxia	Cognitive decline	No
**48**	29	17	M	43	51	Gait instability, motor impersistence	No	No
**49**	29	17	M	57	61	Gait instability, generalised chorea	Mild cognitive decline	No
**50**	29	17	M		33	Choreic movements	No	No
**51**	29	17	U		20	Involuntary movements, tics	No	Obsessive-compulsive disorder
**52**	29	17	U		72	Gait instability, choreic movements	No	No
**53**	29	17	M	74	76	Choreic movements	No	No
**54**	29	19	F	60	81	*not available*		
**55**	29	23	M	73	74	Choreic movements, tremor	No	No
**56**	29	23	F	41	46	No	No	Depression, altered sensory perception
**57**	29	23	U		26	Choreic movements	No	No
**58**	30	15	M	75	82	Involuntary movements, myoclonia	Dementia	Agressive behaviuor
**59**	30	16	F	35	41	Chroreic movements	No	No
**60**	30	17	F	66	68	*not available*		
**61**	30	17	M	64	69	Involuntary movements, dystonia, parkinsonism	No	No
**62**	30	18	U		61	Involuntary movements, tics	No	Adjustment disorder
**63**	30	19	M		29	No	No	Schizophrenia
**64**	30	20	U		38	No	No	Psychiatric disorder, uninhibited behavior
**65**	30	26	U		63	No	Cognitive decline	No
**66**	30	28	M	27	32	Choreic movements	Mild cognitive decline	Psychiatric disorder, uninhibited behavior
**67**	30	30	F	69	71	Choreic movements	Cognitive decline	Psychiatric disorder
**68**	30	30	F	65	65	Oro-bucal dyskinesia	No	Dysthymia
**69**	31	12	M	81	82	Chroreic movements	Cognitive decline	No

**70**	31	15	F	70	73	Involuntary movements	No	No
**71**	31	19	U	40	40	Gait instability	No	No
**72**	31	23	M		32	No	Cognitive decline	No
**73**	32	13	F	43	47	No	No	Depression, perseverative behaviour
**74**	32	17	M	68	71	Unspecified motor symptoms	Mild cognitive decline	Depression
**75**	32	17	M	69	73	Muscle weakness	Dementia	No
**76**	32	19	M	41	41	Dystonia	No	No
**77**	32	20	M	71	73	Gait instability	No	Depression and irritabiility
**78**	32	26	U		86	Choreic movements	No	No
**79**	33	15	M		49	Choreic movements	No	No
**80**	33	16	U		80	Involuntary movements, progressive tics	No	No
**81**	33	16	M		73	Walking difficulties	No	No
**82**	33	17	M	60	61	*not available*		
**83**	33	17	M	53	56	Unspecified motor symptoms	Cognitive decline	Irritability, perseverative thinking
**84**	33	20	U		25	Involuntary movements	No	no
**85**	35	15	F		46	Involuntary movements, tics	No	Anxiety, depression
**86**	35	15	U		77	Chroreic movements	No	No
**87**	35	17	F		78	No	Dementia	No
**88**	35	17	U		50	Ataxia	No	No
**89**	35	18	F	75	81	Parkinsonism	No	No

3. Supplementary Figures

3.1. **Supplementary Fig. 1** Flow diagram illustrating the study populations. For the genotype study, Miseq sequencing was performed in 164 HD subjects and 191 intermediate allele (IA) carriers (symptomatic, unknown phenotype and controls). Of them, we quantified the ratio of CAG expansions in 337 alleles (see **Supplementary Methods**). For the phenotype study, age at onset was available in 242 HD subjects and 49 IA carriers, while data on the type of neurocognitive symptoms was obtained in 82 IA carriers (see **Supplementary Table 3**). For the genotype-phenotype study of IAs, sequencing results and age at sampling data, with or without clinical information, were available in 135 cases. They included 78 individuals with neurocognitive symptoms, 9 individuals with unknown phenotype and 46 population controls: 12 ascertained from population-based cohorts and 34 among asymptomatic non-HD family members (presymptomatic cases carrying an IA, non-related to the family HD chromosome, and married-in subjects).

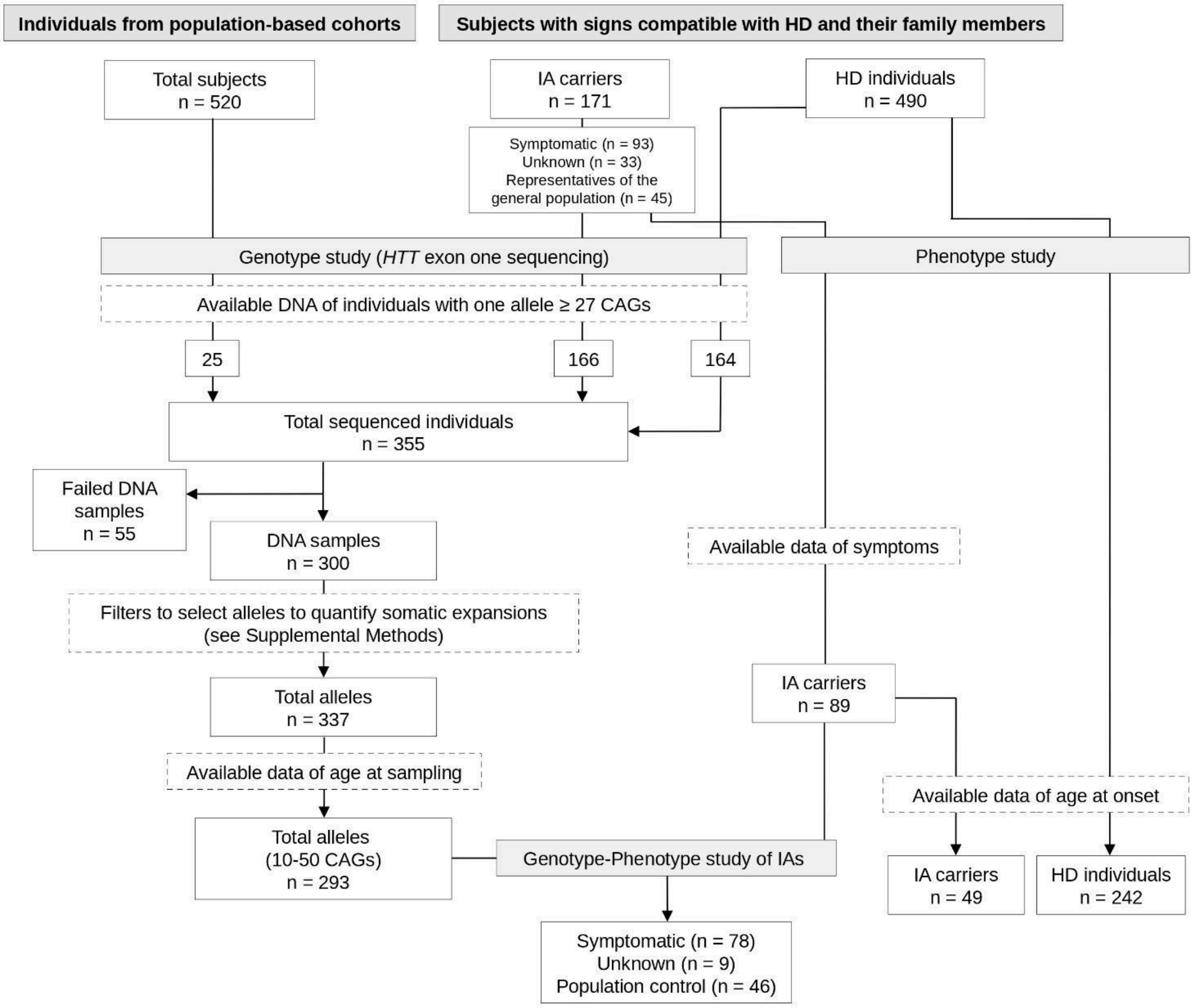

3.2. **Supplementary Fig. 2** Ratio of CAG expansions in intermediate alleles classified by their neurocognitive phenotype into: population control (n=46), symptomatic (n=78) and of unknown phenotype (n=9).

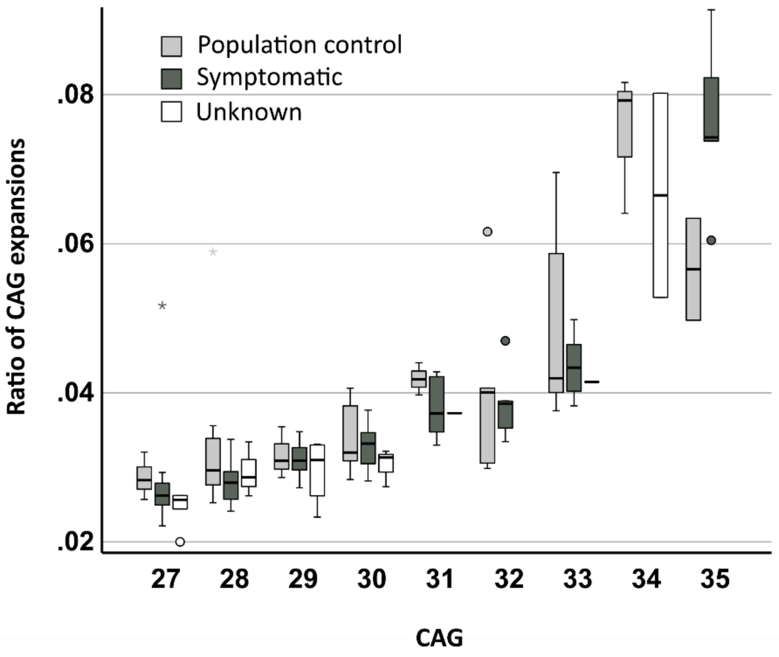

3.3. Supplementary Fig. 3 Family pedigrees of symptomatic intermediate allele carriers that belong to HD families A, B and C. Exon one allele structures are summarised with the number of each trinucleotide or hexanucleotide of the following sequence: (CAG)_(CAACAG)_ (CCGCCA)_(CCG)_(CCT). More details on different exon one sequences are described in Ciosi *et al.*(1). Phased allele structures and haplotypes are included for each individual's chromosomes.

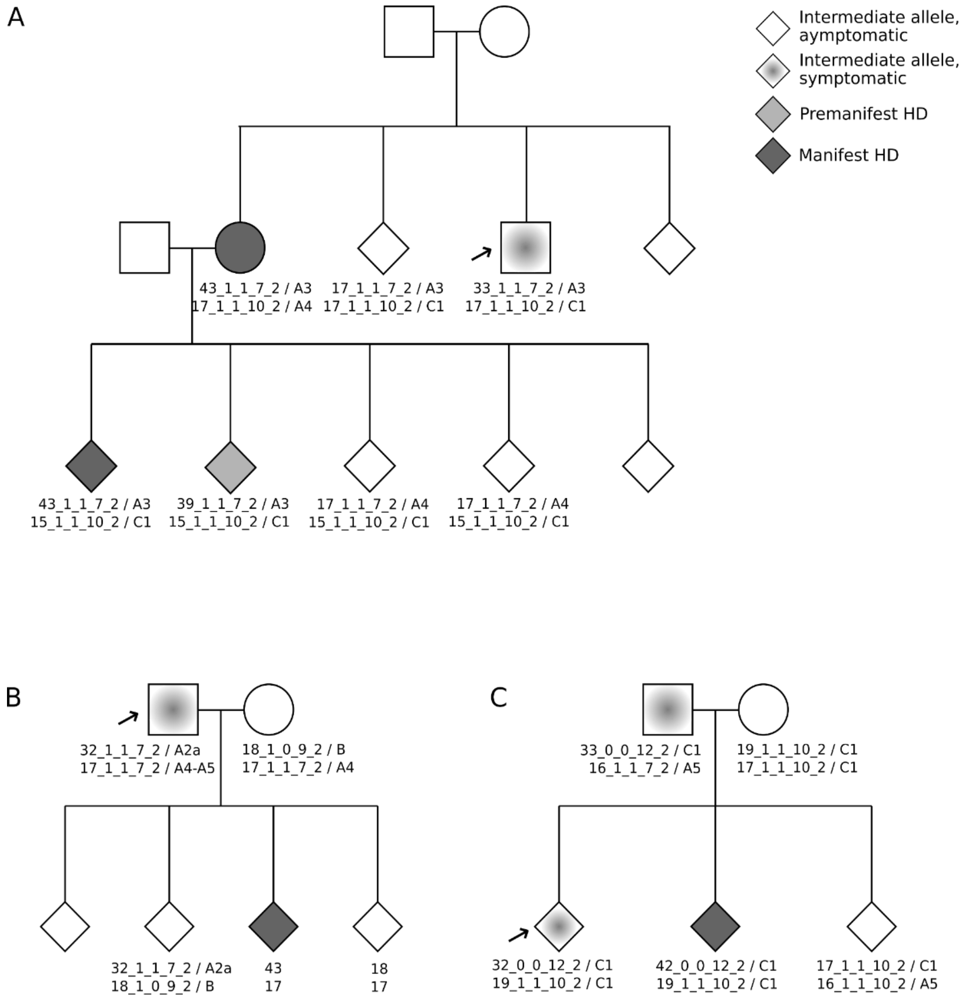

3.4. **Supplementary Fig. 4** Small-pool PCR autoradiographs of different brain and blood tissues that were obtained from an individual with a 33 CAG repeat allele showing neurocognitive symptoms compatible with HD. The panels show small-pool PCR products amplified from 300 pg template DNA of different brain tissues, hybridised with a probe that recognises CAG•CTG variant repeats and Southern blotted. The two main bands observed in each well correspond to 33 and 17 CAG repeats. No large expansions were detected.

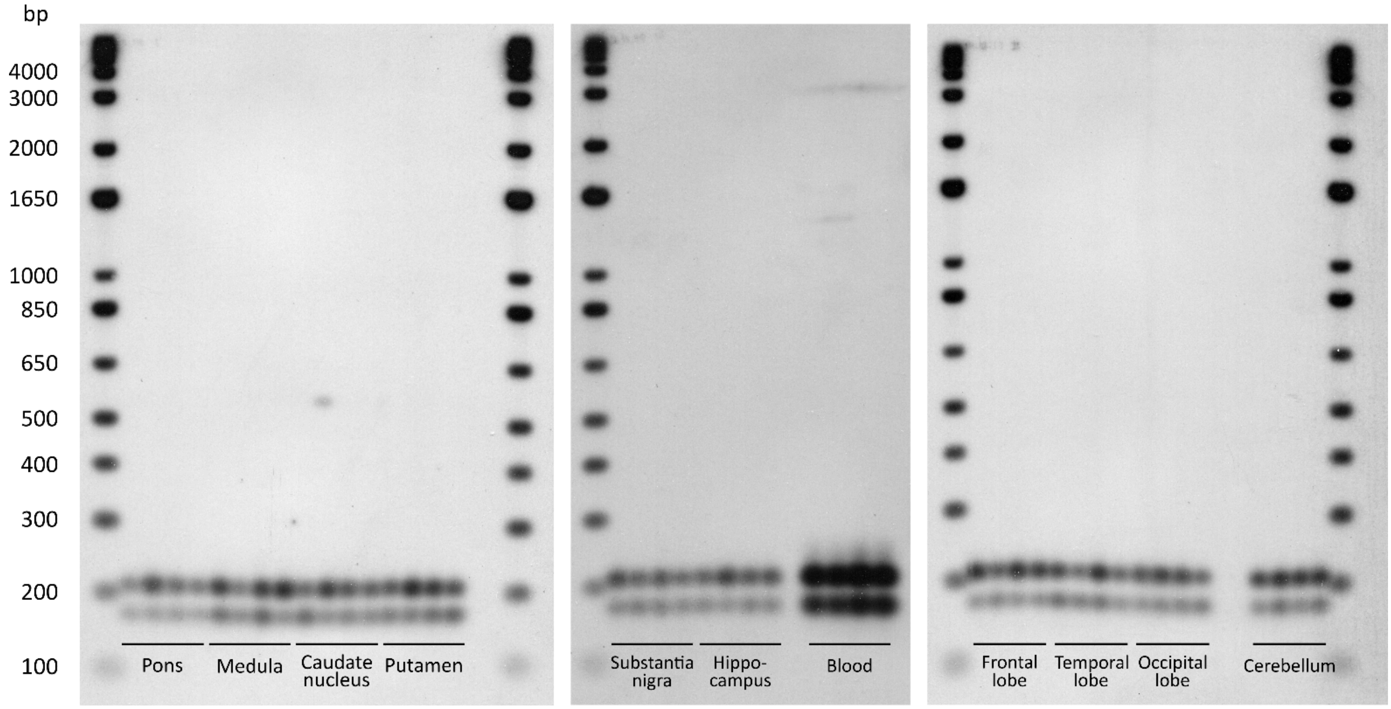

3.5. **Supplementary Fig. 5** Onset of symptoms by the number of CAG repeats in intermediate (green, n=49), reduced-penetrance (orange, n=13) and full-penetrance (blue, n=229) alleles of our cohort, represented along with an additional intermediate allele cohort from the literature (grey, n=35) (2–11). The purple lines indicate the prediction of age at onset by Langbehn *et al*. (12), average (solid line) ± SD (dotted lines).

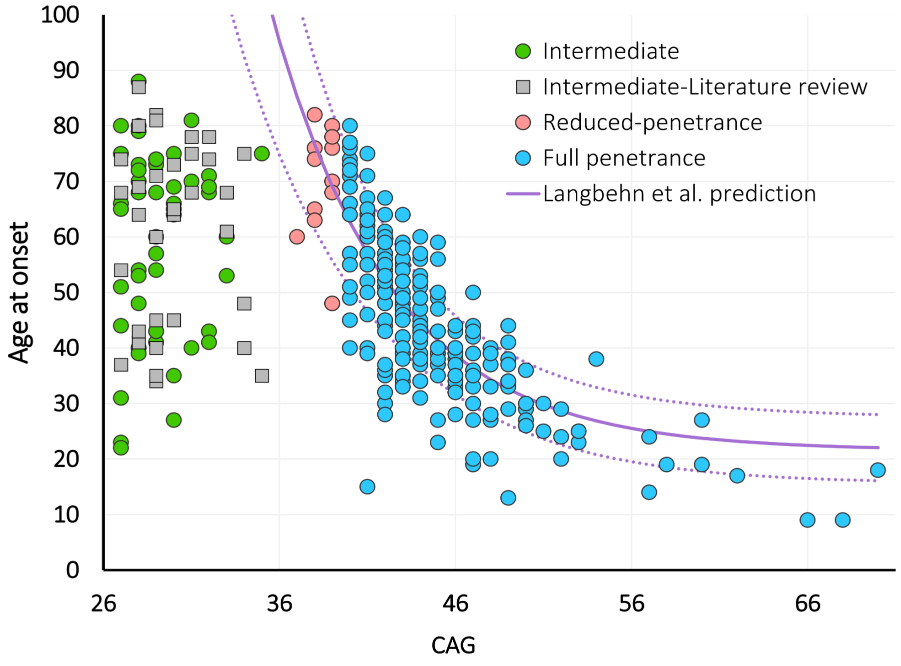

REFERENCES

1. Ciosi M, Maxwell A, Cumming SA, Hensman Moss DJ, Alshammari AM, Flower MD, et al. A genetic association study of glutamine-encoding DNA sequence structures, somatic CAG expansion, and DNA repair gene variants, with Huntington disease clinical outcomes. EBioMedicine [Internet]. 2019 Oct;48:568–80. Available from: http://www.ncbi.nlm.nih.gov/pubmed/31607598

2. Kenney C, Powell S, Jankovic J. Autopsy-proven Huntington's disease with 29 trinucleotide repeats. Mov Disord. 2007 Jan;22(1):127–30.

3. Savitt D, Jankovic J. Clinical phenotype in carriers of intermediate alleles in the huntingtin gene. J Neurol Sci. 2019 Jul 15;402:57–61.

4. Andrich J, Arning L, Wieczorek S, Kraus PH, Gold R, Saft C. Huntington's disease as caused by 34 CAG repeats. Mov Disord. 2008 Apr 30;23(6):879–81.

5. Herishanu YO, Parvari R, Pollack Y, Shelef I, Marom B, Martino T, et al. Huntington disease in subjects from an Israeli Karaite community carrying alleles of intermediate and expanded CAG repeats in the HTT gene: Huntington disease or phenocopy? J Neurol Sci. 2009 Feb 15;277(1–2):143–6.

6. Groen JL, De Bie RM, Foncke EMJ, Roos RAC, Leenders KL, Tijssen MAJ. Late-onset Huntington disease with intermediate CAG repeats: True or false? J Neurol Neurosurg Psychiatry. 2010;81(2):228–30.

7. Ainhi D Ha JJ. Exploring the Correlates of Intermediate CAG Repeats in Huntington Disease. Postgrad Med. 2011;123(5).

8. Squitieri F, Esmaeilzadeh M, Ciarmiello A, Jankovic J. Caudate glucose hypometabolism in a subject carrying an unstable allele of intermediate CAG(33) repeat length in the Huntington's disease gene. Mov Disord [Internet]. 2011 Apr;26(5):925–7. Available from: http://www.ncbi.nlm.nih.gov/pubmed/21370274

9. Ziso B, Larner AJ, Alusi SH. Stuck in the Middle: Huntington's Disease or not Huntington's Disease? J Neuropsychiatry Clin Neurosci [Internet]. 2015 Jan;27(1):e85–6. Available from: http://psychiatryonline.org/doi/abs/10.1176/appi.neuropsych.13110347

10. Garcia-Ruiz PJ, Garcia-Caldentey J, Feliz C, Del Val J, Herranz A, Martínez-Castrillo JC. Late onset Huntington's disease with 29 CAG repeat expansion. Vol. 363, Journal of the Neurological Sciences. Elsevier B.V.; 2016. p. 114–5.

11. Jot S. Parkinsonism with a hint of huntington's from 29 cag repeats in HTT. Brain Sci. 2019;9(10).

12. Langbehn D, Brinkman R, Falush D, Paulsen J, Hayden M. A new model for prediction of the age of onset and penetrance for Huntington's disease based on CAG length. Clin Genet [Internet]. 2004 Mar 12 [cited 2022 May 23];65(4):267–77. Available from: https://onlinelibrary.wiley.com/doi/10.1111/j.1399-0004.2004.00241.x
